# Loss of rapid eye movement atonia in rapid eye movement sleep behaviour disorder and narcolepsy

**DOI:** 10.1111/jsr.14322

**Published:** 2024-08-21

**Authors:** Franziska Edlinger, Marion Böck, Stefan Seidel, Karin Trimmel

**Affiliations:** ^1^ Department of Neurology Medical University of Vienna Vienna Austria

**Keywords:** insomnia, narcolepsy, parasomnias, rapid eye movement sleep, rapid eye movement sleep behaviour disorder

## Abstract

A reduction of physiological muscle atonia during rapid eye movement sleep is characteristic in patients with rapid eye movement sleep behaviour disorder, however, it can also be found in narcolepsy patients. We evaluated rapid eye movement sleep associated electromyographic activity to set cut‐off values of rapid eye movement sleep without atonia, differentiating rapid eye movement sleep behaviour disorder and narcolepsy patients from controls to enable more precise future diagnostic criteria for these disorders. We retrospectively analysed polysomnography recordings of 16 rapid eye movement sleep behaviour disorder patients, 15 narcolepsy patients, and 19 controls. The combination of phasic and tonic electromyographic activity was recorded in the mentalis and tibialis anterior muscles and analysed in 3 second miniepochs. The cut‐off value for a diagnosis of rapid eye movement sleep behaviour disorder was 17.07% (100% sensitivity, 94.7% specificity, area under the curve 0.997). For the diagnosis of narcolepsy, we yielded a cut‐off value of 8.4% (86.4% sensitivity, 68.4% specificity, area under the curve 0.850). Rapid eye movement sleep without atonia significantly (*p* = 0.046) increased in the second night half in rapid eye movement sleep behaviour disorder patients, while it remained moderately increased in the narcolepsy group. Polysomnographic evaluation proves significantly higher rates of rapid eye movement sleep without atonia in rapid eye movement sleep behaviour disorder than in narcolepsy patients, allowing differentiation from controls with high sensitivity and specificity. An increase throughout the night is characteristic for rapid eye movement sleep behaviour disorder, whereas a consistent elevation is typical in narcolepsy patients.

## 1 INTRODUCTION

REM‐sleep behaviour disorder (RBD) is a parasomnia characterised by loss of physiological muscle atonia during REM sleep (REM sleep without atonia, RWA) (American Academy of Sleep Medicine, 2014). A lack of muscle inhibition during the REM sleep stage is associated within dream enactment such as vocalisations and motor behaviours, leading to disrupted sleep and even injuries in the patient or their bed partner (Iranzo et al., 2009). RBD is diagnosed as isolated (iRBD) or secondary to a neurodegenerative disease, however, many patients diagnosed with iRBD develop a synucleinopathy such as Parkinson's disease, multiple system atrophy (MSA), or Lewy body dementia (LBD) later in life (Frauscher et al., 2012; Iranzo et al., 2009; Postuma et al., 2009). The risk of developing a neurodegenerative disease increases with time after RBD diagnosis, leading to the consideration that “isolated” RBD might rather be a precursor of a neurodegenerative disease (Iranzo et al., 2009).

As defined by the ICSD‐3, a clinical history of dream enactment or episodes of REM‐sleep without atonia (RWA) in polysomnography must be present for the diagnosis of RBD (American Academy of Sleep Medicine, 2014). Diagnosis only through clinical history of REM‐sleep related movement might be imprecise, as identifying these behaviours solely through sleep history is challenging. A previous study described moderate interobserver reliability for the diagnosis of RBD using only patient interviews for diagnosis, as there was a high level of disagreement among specialists in defining dream associated movements (Vignatelli et al., 2005). Other sleep disturbances such as obstructive sleep apnea can also lead to similar motor behaviour like those typical for RBD, which must be clearly delineated, however, as these only occur during respiratory‐related arousals (Iranzo & Santamaría, 2005). Therefore polysomnographic documentation of RWA is important for distinguishing forms of motor behaviour and ensuring a highly sensitive and specific diagnosis of RBD.

In patients with narcolepsy, RWA is also frequently observed, with a predominance in narcolepsy with cataplexy (type 1), where RWA may occur in over 60% of cases (Antelmi et al., 2020), while symptoms are usually milder than in RBD patients (Nevsimalova et al., 2013; Nightingale et al., 2005). Narcolepsy with cataplexy is a central disorder of hypersomnolence, characterised by an imbalance in the sleep–wake cycle due to a reduction in hypocretin‐1 producing neurons in the hypothalamus (Young & Heidbreder, 2018). Loss of hypocretin‐1 neurons has further been suggested to contribute to RWA in narcolepsy with cataplexy due to projections to motor neurons in the spinal cord (Knudsen et al., 2010). However, RWA may occur both in narcolepsy type 1, which is linked to hypocretin deficiency, as well as in narcolepsy type 2, where hypocretin levels are mostly normal and cataplexy is not present. With RBD occurring in all types of narcolepsy and even having been described as a possible symptom precursor before diagnosis, its exact pathophysiological role remains unclear (Antelmi et al., 2020; Bonakis et al., 2009). RWA in narcolepsy patients differs in expression and nightly distribution from RBD patients, with episodes being generally milder and patients expressing less violent behaviour. While RWA episodes occur predominantly in the second half of the night in RBD patients, episodes in narcolepsy patients tend to appear evenly throughout the course of the night (Antelmi et al., 2019; Antelmi et al., 2020; Cipolli et al., 2011; Franceschini et al., 2011). Further, motor behaviour patterns differ not only from iRBD patients but also within narcolepsy patients affected by RWA, suggesting that RWA in narcolepsy is an independent phenomenon and needs further investigation to analyse its nature and enable differentiation from RWA in iRBD (Antelmi et al., 2020).

Since the release of the ICSD‐2, polysomnography must be performed for diagnosis, however, standardised muscle groups and specific cut‐off values to differentiate RBD and narcolepsy patients from healthy individuals are yet to be determined (Frauscher et al., 2012). A previous study comparing REM‐sleep associated EMG activity in the submental muscles has found that RBD‐ and narcolepsy patients had significantly higher tonic and phasic EMG activity than controls, however, differences in EMG activity between RBD‐ and narcolepsy patients did not reach statistical significance (Khalil et al., 2013). In another study, RWA was significantly more prevalent in RBD and narcolepsy patients compared with controls, but the narcolepsy group still had a significantly lower percentage than the RBD group (Dauvilliers et al., 2007).

Regarding muscle groups, the mentalis muscle has frequently been used to study EMG activity during REM sleep and cut‐off values have been suggested for RBD diagnosis (Frauscher et al., 2012). As the mentalis muscle might lead to false‐positive results from breathing and snoring artefacts and is not involved when limb movements occur, the inclusion of additional muscles such as the tibialis anterior‐ or upper limb muscles has been recommended (Fernández‐Arcos et al., 2017; Frauscher et al., 2008; McCarter et al., 2014; McCarter et al., 2017).

Despite the recognition of RWA as a common aspect of both RBD and narcolepsy, standardised criteria to interpret elevated muscle activity during REM sleep as well as validated cut‐off values for differentiating RBD and narcolepsy from controls are lacking.

A recent systematic review (Puligheddu et al., 2023) concluded that while quantification of RWA is supported in the diagnostic workup of disorders such as RBD or narcolepsy, more studies on this topic are warranted, particularly as definitive cut‐off values of RWA have been inconsistent. To our knowledge, only one previous study (Khalil et al., 2013) included both RBD as well as narcolepsy patients to define diagnostic cut‐off values of RWA compared with controls in a small sample. The present study aimed to validate diagnostic thresholds of REM‐associated EMG activity in both RBD and narcolepsy compared with controls using standardised scoring methods (Frauscher et al., 2012), and to additionally include the analysis of overnight distribution of RWA as a potential diagnostic marker.

## 2 METHODS

### 2.1 Subjects

Polysomnographic recordings used for the present study were obtained from patients admitted to the sleep laboratory at the Medical University of Vienna between January 2012 and September 2021 for retrospective analysis. If more than one night of PSG was performed, only the first available study was included for consistency. All patients with a diagnosis of RBD or narcolepsy were considered, and patients with insomnia or NREM parasomnia were used as the control group. None of the narcolepsy patients had a history of RBD episodes. Diagnosis of RBD, narcolepsy, insomnia, and NREM‐parasomnias was made according to ICSD‐3 (American Academy of Sleep Medicine, 2014) by neurologists specialised in sleep disorders (KT, SS). Eleven/16 (68.8%) RBD patients were classified as having isolated RBD, while 5/16 (31.3%) had been diagnosed with Parkinson's disease before the study. Patients with insufficient quality of PSG data or too short/fragmented REM sleep (≤10 min) were excluded. All RBD‐ and control patients were over the age of 18 years. In the narcolepsy group, the age limit was set at 15 years to reach a sufficient patient number. Control patients taking antidepressant medication were excluded from the study due to the known side effect of antidepressants provoking dream‐enacting behaviours and loss of REM sleep atonia (Postuma et al., 2013). After applying exclusion criteria to the initial study group of 62 patients, a total of 50 patients were included, of which 16 were RBD patients, 15 narcolepsy patients, and 19 controls.

Chart review included the patients sleep history, age, sex, body mass index (BMI), co‐existing medical conditions, current medication, and subjective sleep parameters (Pittsburgh Sleep Quality Index [Buysse et al., 1989] and Epworth Sleepiness Scale [Arnulf et al., 2015] scores). Demographic and clinical characteristics are listed in Table 1.

**TABLE 1:** Demographic, clinical characteristics, and REM‐sleep associated EMG activity in RBD, narcolepsy patients, and controls.

		RBD (1) *n* = 16	Narcolepsy (2) *n* = 15	Controls (3) *n* = 19	*p*			
					Total	1 versus 2	1 versus 3	2 versus 3
Age (median IQR; year)		68.5 (14)	25 (18)	30 (10)	<0.001	<0.001	<0.001	0.203
Gender	Male (*n*/%) Female (*n*/%)	15/93.8 1/6.3	9/60 6/40	6/31.6 13/68.4	<0.001	0.037	<0.001	0.165
BMI (mean ± SD)		25.2 ± 3.3	27.6 ± 6.3	22.5 ± 3.1	0.006	0.398	0.229	0.004
Patients using concurrent medication	Antidepressants (*n*/%)	4/25	3/20	0/0	0.05	0.539	0.035	0.076
	Antiparkinsonian (*n*/%)	7/43.8	0/0	0/0	<0.001	0.004	0.002	n.a.
Mentalis^a^		41.4 (30.4)	14.8 (16.8)	4.3 (5.3)	<0.001	0.002	<0.001	0.022
Tibialis ant^a^		15.4 (15.5)	6.4 (12.6)	2.8 (2.7)	<0.001	0.035	<0.001	0.008
m&t^a^		51.8 (29.7)	17.6 (16)	5.9 (6.7)	<0.001	0.008	<0.001	0.005

*Note*: Data are represented as mean ± SD, median (IQR) or frequency/percentage according to data distribution.

Abbreviations: BMI, body‐mass‐index; m&t, m. mentalis and m. tibialis anterior; RBD, REM‐sleep behaviour disorder, tibialis ant, m. tibialis anterior.

^a^ Data in percent (%).

### 2.2 Polysomnography

Polysomnography included electroencephalography (F3, C3, O1 with M2 as reference electrode), electrooculography (vertical and horizontal eye movements), single‐channel electrocardiography, respiratory recording (nasal pressure transducer and oronasal airflow sensor, piezoelectric snore sensor, transcutaneous oximetry, respiratory movements from induction plethysmography, and sensors for body positioning), video documentation, and surface electromyogram (EMG) from the mentalis and tibialis anterior muscles.

Sleep, limb movements (LM), and arousals were scored according to AASM (Berry et al., 2020). EMG activity was recorded with bipolar surface electrodes with a low‐pass filter at 100 Hz, high‐pass filter at 10 Hz, and a sampling rate of 500 Hz. Amplification was 5 μV/mm for REM‐related muscle activity, and 10 μV/mm for isolated LM and periodic LM. Impedance of EMG electrodes was <5 kΩ (chin), respectively <10 kΩ (leg). All arousal‐ or artefact‐related increases in EMG tone were excluded from quantitative scoring in line with previous suggestions (Frauscher et al., 2012). To prevent possible influence of apnea episodes on the detection of EMG activity, all patients with an AHI > 10 were excluded from the study. Muscle activity during REM sleep was subdivided into 3 second miniepochs and categorised in the presence or absence of EMG activity (defined by EMG amplitudes > twice that of the background EMG activity) by a single polysomnographic technologist (MB) with >20 years of scoring experience. Epochs of both tonic (scored only in mentalis muscle, defined as EMG activity in >50% of a 30 s epoch) and phasic muscle activity (any EMG activity lasting between 0.1 and 5 s), combined as “any” were recognised as the presence of EMG activity, which were summarised as a percentage in accord with the existing literature (Frauscher et al., 2012).

To evaluate the distribution of EMG activity throughout the night, its temporal occurrence was assigned to the first or second half of total sleep time, as in a previous study (Cipolli et al., 2011). Differences in REM‐sleep associated muscle activity between the night halves within each patient and across the patient groups were evaluated.

### 2.3 Statistics

SPSS software (version 27.0.1.0, IBM Corp., Armonk, N.Y., USA) was used for statistical analysis. Descriptive statistics were expressed as mean/standard deviation or median/interquartile range, while discrete data were given as frequencies/percentage. One‐way analyses of variance (ANOVA) or Kruskal‐Wallis tests were used to test mean or rank differences (according to distribution of data) among the subgroups regarding EMG activity and polysomnographic data. Receiver‐operating‐characteristics (ROC) curves were calculated for combined phasic and tonic EMG activity in the mentalis muscles, the tibialis anterior muscles, and their combination. Diagnostic cut‐off thresholds were identified, combining the highest sensitivity and specificity for a diagnosis of RBD or narcolepsy.

The non‐parametric Friedmann test was used to assess differences in temporal distribution of REM‐sleep in the patient groups. Age differences were tested using the Kruskal‐Wallis test, for gender differences, Fisher‐exact test was performed. To correct for age as a confounding variable regarding EMG activity and its nightly distribution, the non‐parametric analysis of covariance (Quade's ANCOVA) was used.

## 3 RESULTS

### 3.1 Demographic data

Demographic data are presented in Table 1. Six/16 (37.5%) RBD patients were taking sleep‐specific medicine (5 melatonin, 1 trazodone), 4/16 (25%) were on antidepressants. Seven/16 (43.8%) were taking antiparkinsonian medication, of which 5 had been diagnosed with Parkinson's disease before. Twelve/15 (80%) of the narcolepsy subgroup were diagnosed with type 1 narcolepsy and 3/15 (20%) with type 2. Ten/15 (66.7%) patients were taking sleep specific medicine (5 modafinil, 2 sodium oxybate, 2 rotigotine [for PLMs], 1 pitolisant) and 3/15 (20%) were taking antidepressant medication. The control group included 8 insomnia patients, 10 patients with NREM parasomnias, and 1 with catathrenia. Two controls took sleep‐specific medicine (1 brotiozolam, 1 trazodone), none were taking antidepressant or antiparkinsonian medication.

There was a significant age difference among the patient groups (F(2, 47) = 30.67, *p* < 0.001). The RBD patients were significantly older compared with the narcolepsy patients (*p* < 0.001) and controls (*p* < 0.001), while there was no significant age difference between narcolepsy patients and controls (*p* = 0.203). Gender distribution showed a statistically significant male predominance in the RBD group, compared with the narcolepsy group (two‐tailed *p* = 0.037) and the control group (two‐tailed *p* < 0.001). In the narcolepsy group, gender distribution was more balanced with a slight male predominance. In the control group, gender composition showed a female predominance. Narcolepsy patients had the highest mean BMI, followed by RBD patients and lastly control patients (F(2, 47) = 5.74, *p* = 0.006).

### 3.2 PSG characteristics

The PSG data are listed in Table S1. Polysomnography showed that RBD patients had a significantly shorter total sleep time (TST, [H (2) = 14.81, *p* < 0.001]) than narcolepsy patients (*p* = 0.001) or controls (*p* = 0.004). Wake after sleep onset time (WASO, [H (2) = 13.6, *p* = 0.001]) was longer in the RBD group than in the narcolepsy patients (*p* = 0.013) or controls (*p* = 0.002). Sleep efficiency was lower in RBD patients (H (2) = 16.8, *p* < 0.01) than in the narcolepsy patients (*p* = 0.002) and controls (*p* = 0.001).

The RBD patients had the highest amount of stage 1 sleep (H (2) = 15.99, *p* < 0.001), followed by narcolepsy patients (*p* = 0.043) and controls (*p* < 0.001). Stage 3 sleep (H (2) = 18.57, *p* < 0.001) was significantly shorter in the RBD patients than in the narcolepsy patients (*p* < 0.001) or controls (*p* = 0.001).

The total AHI (H (2) = 7.95, *p* = 0.019) was higher in the RBD patients than in the controls (*p* = 0.016), while there was no difference compared with the narcolepsy patients (*p* = 0.227). AHI in NREM sleep (H (2) = 8.17, *p* = 0.017) as well as the arousal index (H (2) = 6.36, *p* = 0.042) were significantly higher in the RBD patients than in the controls (AHI: *p* = 0.020, arousal index: *p* = 0.019). The PLM index (H (2) = 28.95, *p* < 0.001) was significantly higher in the RBD patients compared with the narcolepsy patients (*p* = 0.028) and controls (*p* < 0.001). The narcolepsy patients had a significantly shorter REM latency after sleep onset (H (2) = 8.65, *p* = 0.013), compared with the RBD patients (*p* = 0.017) and the controls (*p* = 0.020).

The RBD patients reported the lowest subjective sleep quality (H (2) = 8.19, *p* = 0.017), differing significantly from the narcolepsy patients (*p* = 0.022), but not from the controls (*p* = 0.083), while the narcolepsy patients stated the shortest subjective sleep latency (H (2) = 11.53, *p* = 0.003), differing significantly from the control group (*p* = 0.002). There was no significant difference in subjective sleep efficiency (H (2) = 3.03, *p* = 0.220), PSQI (H (2) = 0.88, *p* = 0.643) or ESS scores (H (2) = 3.46, *p* = 0.177) among the groups.

### 3.3 EMG activity

**FIGURE 1:**
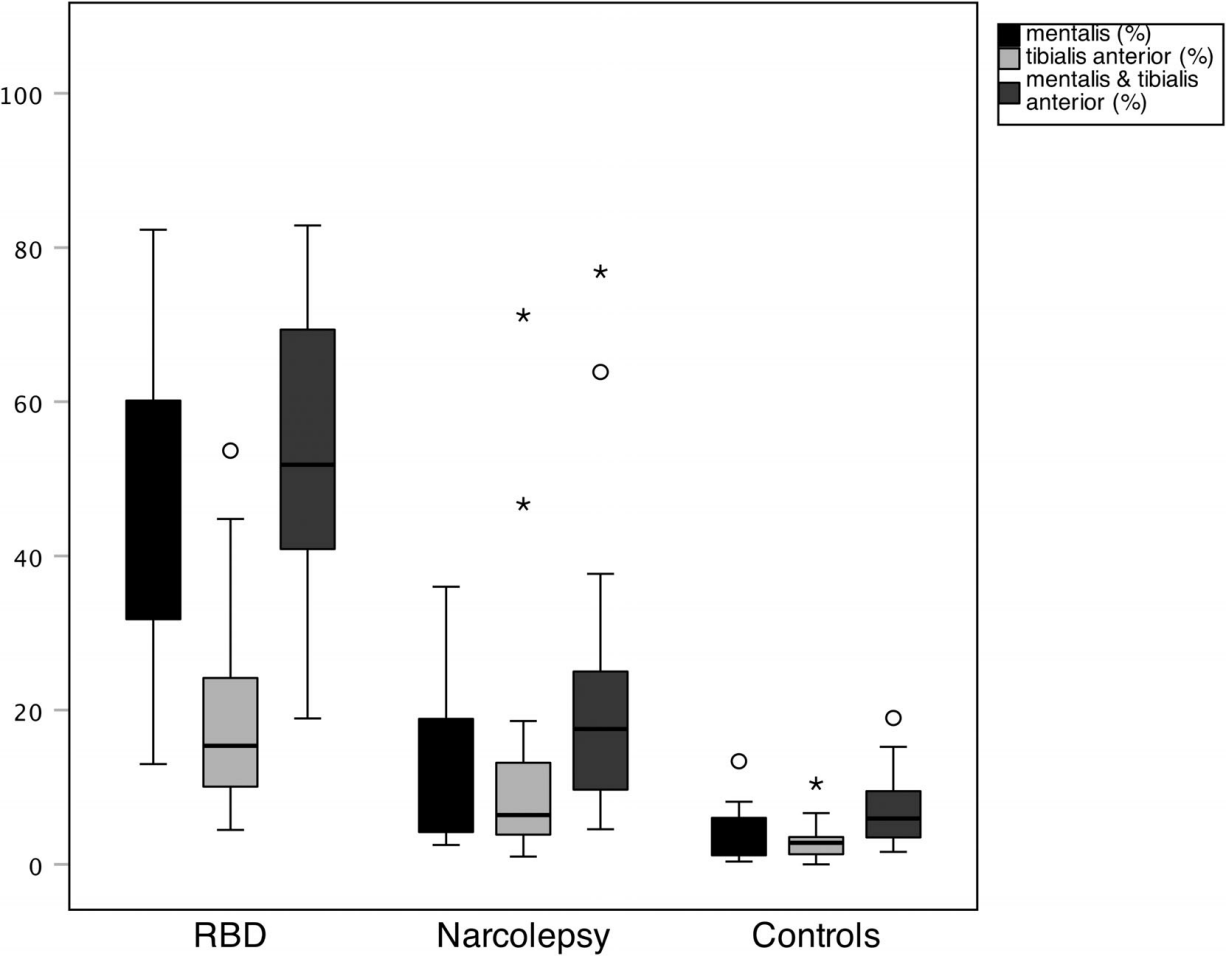
REM‐sleep related EMG activity in the three muscle groups among the subgroups. Boxes show median, 25th and 75th percentiles as horizontal lines, whiskers represent 1.5 times the interquartile rate of the data, circles represent outliers, asterisks represent extreme outliers. RBD, REM‐sleep behaviour disorder, m&t, m. mentalis and m. tibialis anterior.

There was a significant difference in REM‐sleep related EMG activity in the mentalis‐ and tibialis anterior muscles and their combination in all groups (mentalis: H (2) = 32.03, *p* < 0.001; tibialis: (H (2) = 24.59, *p* < 0.001); combined: H (2) = 32.12, *p* < 0.001). REM‐sleep related EMG activity is listed in Table 1 and illustrated in Figure 1. The EMG activity in the mentalis, tibialis anterior muscles, and their combination was significantly higher in the RBD patients than in the controls (all *p*‐values <0.001). Mentalis muscle activity was significantly higher in RBD than in the narcolepsy patients (*p* = 0.005), however, tibialis anterior muscle activity did not show significant differences (*p* = 0.140). Median EMG activity in the muscle combination differed significantly between RBD and narcolepsy patients (*p* = 0.024).

Narcolepsy patients also had higher amounts of EMG activity than the controls. Mentalis muscle activity was higher in the narcolepsy group than in the controls, however, it did not reach statistical significance (*p* = 0.067). Tibialis anterior activity was significantly higher in narcolepsy patients compared with the control group (*p* = 0.024), as well as the muscle combination (*p* = 0.015).

### 3.4 Cut‐off values

Characteristics of the ROC‐curves and cut‐off values for the diagnosis of RBD and narcolepsy versus controls are listed in Table 2.

**TABLE 2:** ROC characteristics in RBD and narcolepsy patients.

	RBD *n* = 16			Narcolepsy *n* = 15		
	m	t	m & t	m	t	m & t
Sensitivity	100%	93.8%	100%	66.7%	73.3%	86.4%
Specificity	94.7%	89.5%	94.7%	73.7%	78.9%	68.4%
Cut‐off	≥ 10.57%	≥ 6.39%	≥ 17.07%	≥ 5.97%	≥ 4.03%	≥ 8.37%
95% CI	0.976–1	0.932–1	0.986–1	0.647–0.953	0.621–0.944	0.717–0.981
AUC	0.993	0.974	0.997	0.800	0.782	0.849

Abbreviations: AUC, area under the curve; CI, confidence interval; m, m. mentalis; m & t, m. mentalis & m. tibialis anterior; RBD, REM sleep behaviour disorder; t, m. tibialis anterior.

### 3.5 RBD versus controls

**FIGURE 2:**
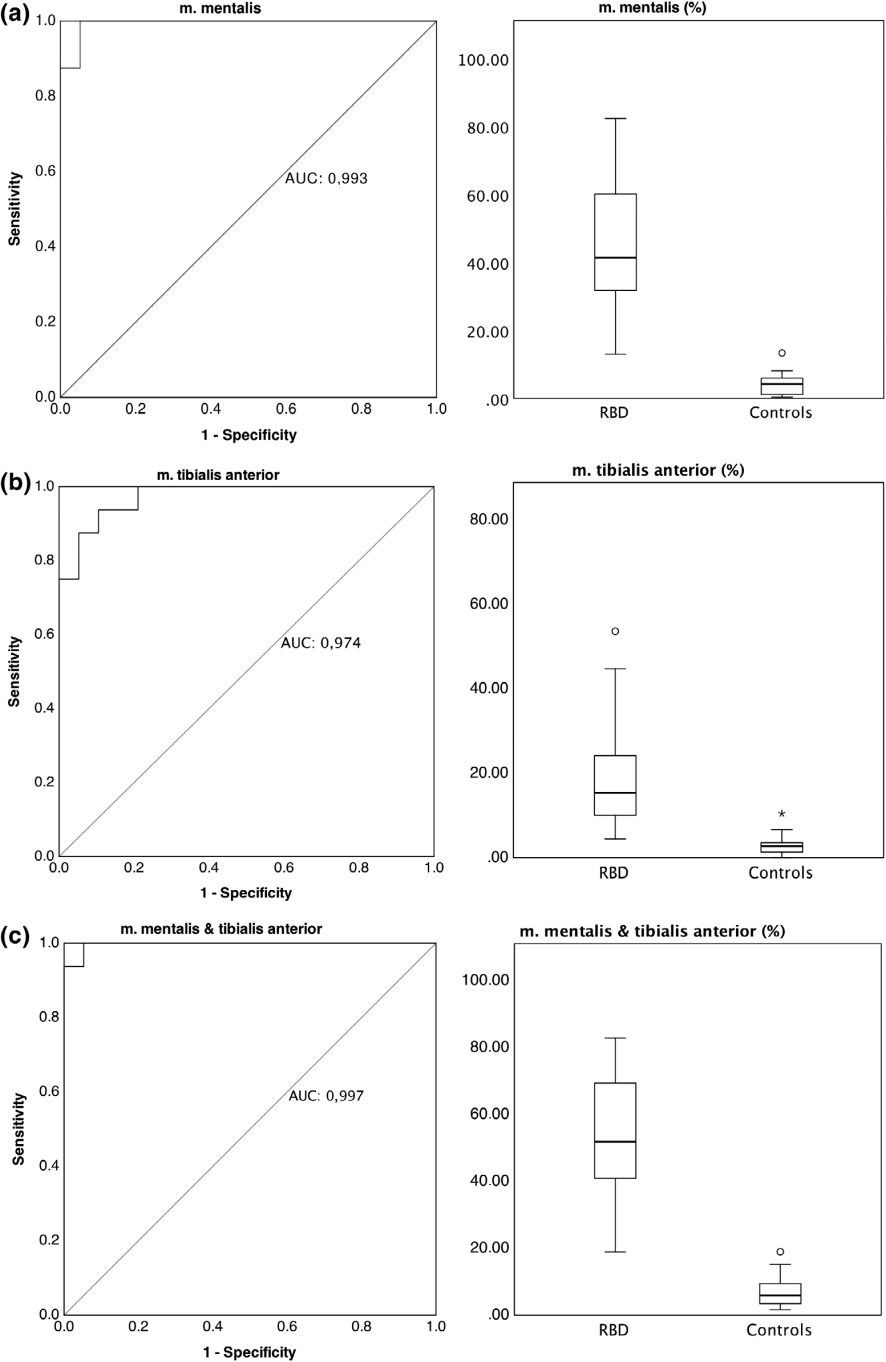
ROC‐curves and boxplot representations of muscle activity in the mentalis (a), tibialis anterior muscle (b), and combination (c) in RBD patients and controls. Boxes show median, 25th and 75th percentiles as horizontal lines, whiskers represent 1.5 times the interquartile rate of the data, circles represent outliers. AUC, area under the curve, RBD, REM sleep behaviour disorder, ROC, receiver‐operating characteristics curve.

For the diagnosis of RBD versus controls, the highest AUC value (0.997) was found for the combination of mentalis and tibialis anterior muscles with a cut‐off value of 17.07% REM‐associated muscle activity, yielding 100% sensitivity and 94.7% specificity (Figure 2, Table 2). Slightly lower values were observed when mentalis and tibialis anterior muscles were analysed separately (Figure 2, Table 2).

### 3.6 Narcolepsy versus controls

**FIGURE 3:**
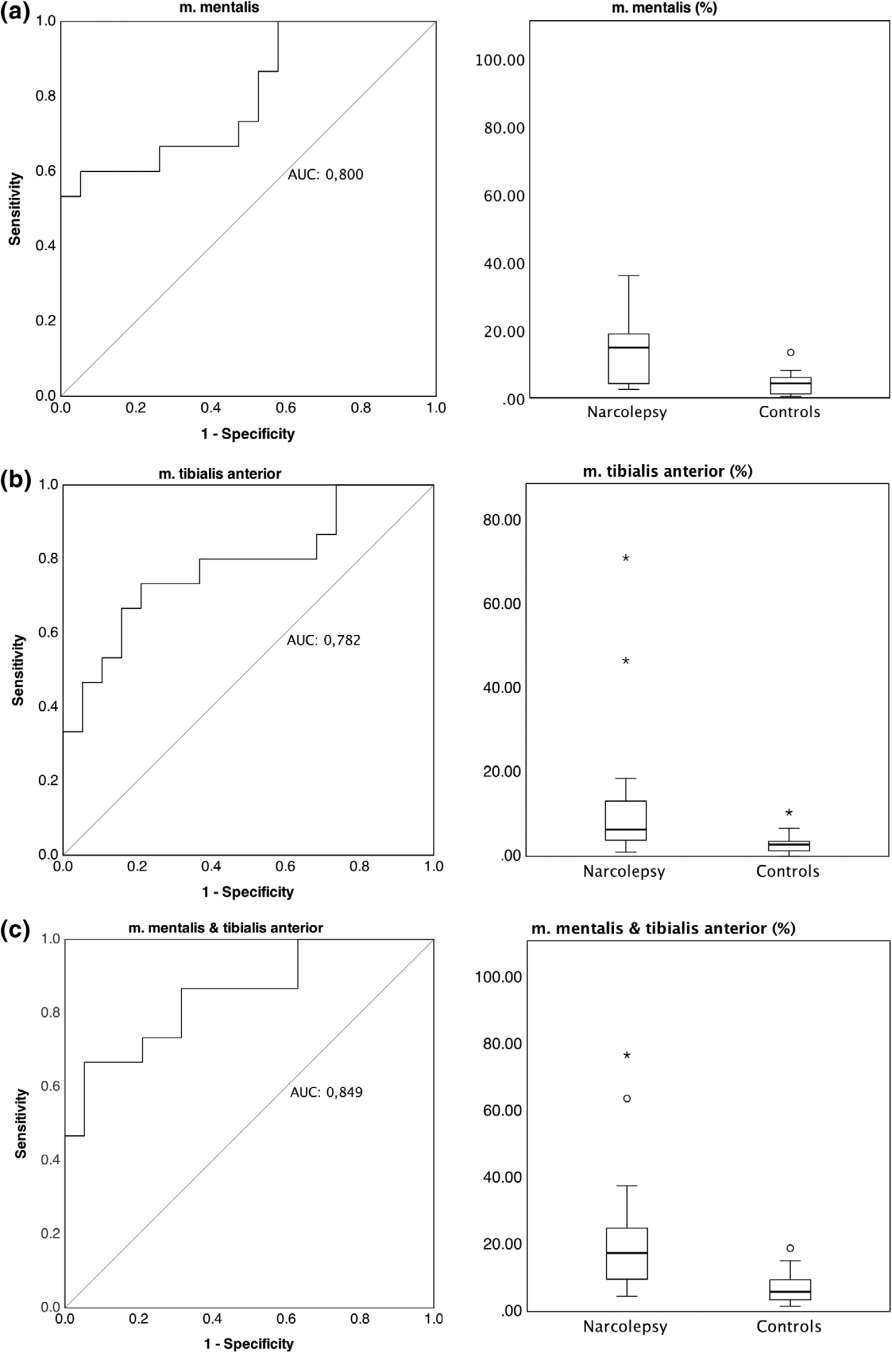
ROC‐curves and boxplot representations of muscle activity in the mentalis (a), tibialis anterior muscle (b), and combination (c) in narcolepsy patients and controls. Boxes show median, 25th and 75th percentiles as horizontal lines, whiskers represent 1.5 times the interquartile rate of the data, circles represent outliers. AUC, area under the curve, ROC, receiver‐operating characteristics curve.

Sensitivity and specificity of cut‐off values for the diagnosis of narcolepsy versus controls were lower than those for RBD. The highest AUC was again found for the combination of mentalis and tibialis anterior muscles (0.849) at a cut‐off value of 8.37% EMG activity with a sensitivity of 86.4% and specificity of 68.4%, with slightly lower values for mentalis muscles (AUC 0.800) and lowest discriminative power in the tibialis anterior muscle analysis (AUC 0.782, Figure 3, Table 2).

### 3.7 Overnight distribution of EMG activity

**FIGURE 4:**
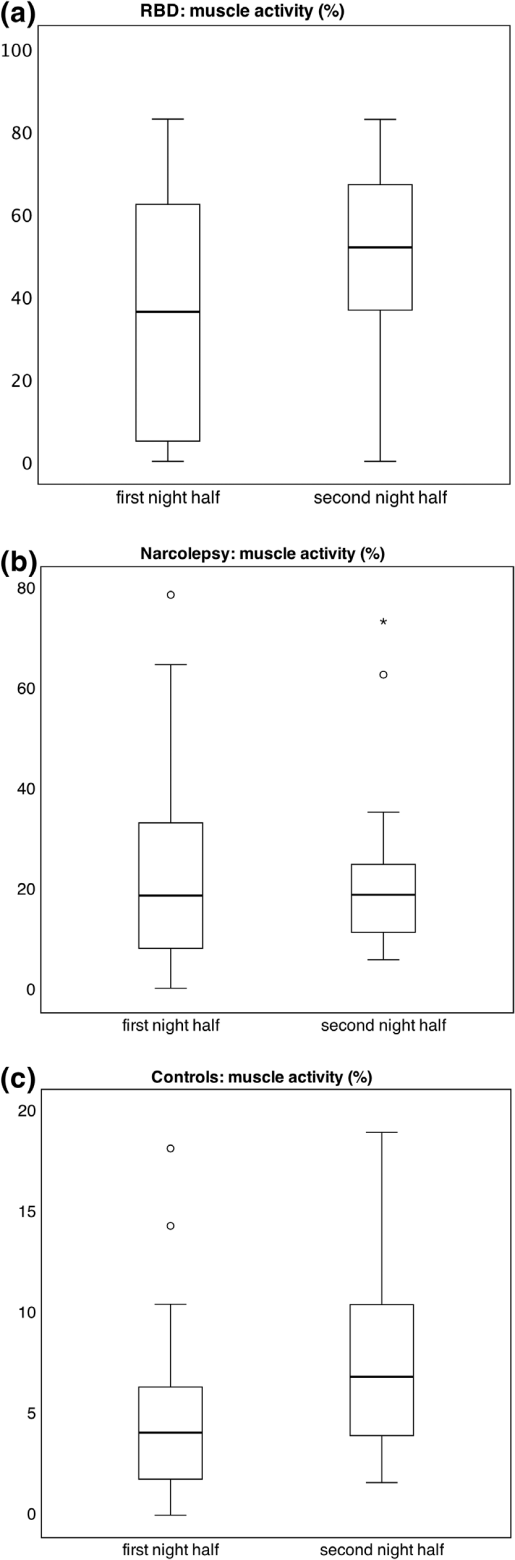
Differences in muscle activity between the night halves in RBD patients (a), narcolepsy patients (b), and controls (c). Boxes show median, 25th and 75th percentiles as horizontal lines, whiskers represent maximum/minimum (RBD patients) or 1.5 times the interquartile rate of the data (narcolepsy/control patients), circles represent outliers, asterisks represent extreme outliers. RBD, REM‐sleep behaviour disorder.

The EMG activity during REM sleep in the mentalis and tibialis anterior muscle combination was compared between the first and second half of the night, as listed in Table 3. The EMG activity increased significantly from the first to the second half of the night in RBD patients (H (1) = 4.00, *p* = 0.046) and in controls (H (1) = 15.21, *p* < 0.001, Figure 4, Table 3). On the contrary, the EMG rates in narcolepsy patients did not change significantly between the first and second half of the night (H (1) = 0.60, *p* = 0.439; Figure 4, Table 3).

**TABLE 3:** Overnight distribution of REM‐associated EMG activity in the night halves in RBD, narcolepsy patients, and controls.

	RBD *n* = 16	Narcolepsy *n* = 15	Controls *n* = 19
% EMG activity first half of the night^a^	36.2 (60.7)	18.9 (34)	4.1 (5)
% EMG activity second half of the night^a^	51.8 (32.4)	19.1 (13.9)	6.9 (7.6)
*p*	0.046	0.439	<0.001

*Note*: Data are represented as median and interquartile range (IQR).

^a^ Data in percent (%).

### 3.8 Correlations

No significant correlations were found between demographic parameters (age, gender, BMI) and EMG activity in the mentalis or tibialis anterior muscles or their combination in any of the patient groups (all *p* > 0.05).

### 3.9 Sensitivity analyses

To control for confounding effects of antidepressant medication, we performed a subanalysis excluding patients taking antidepressants (including trazodone) or brotizolam. Details of this analysis are provided in Tables S2 and S3 as well as in Figures S1 and S2. There was still a significant difference between mentalis and tibialis anterior muscle activity as well as their combination between the groups (mentalis: H (2) = 23.89, *p* < 0.001; tibialis: (H (2) = 18.07, *p* < 0.001); combined: H (2) = 25.00, *p* < 0.001).

The analyses of the ROC curves also only showed minor differences in the subanalysis compared with the original analysis. For the diagnosis of RBD versus controls, the cut‐off value for the mentalis muscle was ≥10.57% with a sensitivity of 100% and specificity of 94.4%, the cut‐off value for the tibialis anterior muscle was ≥6.39% with a sensitivity of 90.9% and a specificity of 88.9% and the muscle combination had a cut‐off value of ≥17.07% with a sensitivity of 100% and a specificity of 94.4%.

For the diagnosis of narcolepsy, the cut‐off value for the mentalis muscle was ≥5.97% with a sensitivity of 61.5% and a specificity of 72.2%, the cut‐off value for the tibialis anterior was at ≥4.03% with a sensitivity of 69.2% and a specificity of 77.8% and the cut‐off value for the muscle combination had a cut‐off of ≥8.37% with a sensitivity of 84.6% and a specificity of 66.7%.

Additionally, Quade's covariance analysis was performed to control for age as a confounding factor of EMG activity among the groups, giving similar results to the original calculations. The difference in EMG activity between the RBD group and controls remained significant in the mentalis‐ (*p* < 0.001), tibialis anterior muscles (*p* = 0.003) and their combination (*p* < 0.001). In the narcolepsy group, the EMG rates were still significantly different from controls in all muscle groups (mentalis: *p* = 0.002, tibialis anterior: *p* = 0.002, combination: *p* < 0.001). The difference in EMG activity between RBD‐ and narcolepsy patients was no longer significant (mentalis: *p* = 0.235, tibialis anterior: *p* = 0.966, combination: *p* = 0.641).

Differences in EMG activity between the night halves were also explored using age as a covariate, showing significant differences among all muscle groups in RBD‐, narcolepsy‐patients, and controls (mentalis: *p* = 0.036, tibialis anterior: 0 = 0.001, combination: *p* = 0.013).

## 4 DISCUSSION

Quantifying REM sleep‐associated EMG activity is a valuable tool for understanding and diagnosing RBD and for differentiating from other sleep disorders and healthy individuals. This study investigated REM‐sleep associated EMG activity of the mentalis and tibialis muscles and their combination as well as their distribution throughout the night and provided highly discriminative cut‐off values for EMG activity to differentiate RBD and narcolepsy patients from controls. As described previously (Dauvilliers et al., 2007), EMG activity was highest in the RBD group, followed by narcolepsy patients, who still had significantly higher rates of RWA compared with controls. The diagnostic accuracy to differentiate between RBD or narcolepsy and controls was highest when combining muscle activity from the mentalis and tibialis anterior muscles. RWA levels are known to be physiologically higher in older men, which corresponds to our RBD subgroup, predominantly consisting of male and significantly older patients than the other patient groups (Feemster et al., 2019). However, differences in EMG activity remained significant between RBD patients and controls and narcolepsy patients and controls after controlling for age. The difference between RBD patients and narcolepsy patients was no longer significant, which might be attributable to the small sample size, limiting statistical power.

In the RBD group, we could confirm the high discriminative power of EMG activity, with a cut‐off value of ≥17.07% allowing us to differentiate between RBD and controls with 100% sensitivity and 94.7% specificity, which was lower compared with similar studies, ranging in cut‐off values between 27% and 46.4% in previous studies, with AUC ranging from 0.95 to 1.000 (Figorilli et al., 2020; Frauscher et al., 2012; McCarter et al., 2014; McCarter et al., 2017). In a 2013 study, however, cut‐off values for RBD diagnosis were considerably lower, with ≥3.17% tonic (87% sensitivity, 100% specificity, AUC 0.9) and ≥1.22% phasic (100% sensitivity, 100% specificity, AUC 1) EMG activity (Khalil et al., 2013). We observed comparably high sensitivity and specificity values for the combination of mentalis and tibialis anterior muscles with an AUC of 0.997.

In the narcolepsy group, a cut‐off value of ≥8.37% differentiated patients from controls at a sensitivity of 86.4% and a specificity of 68.4%. Khalil et al. (2013) investigated RWA cut‐off values in adult narcolepsy patients and suggested lower cut‐offs (≥2.0% phasic and ≥1.48% tonic activity in the submentalis muscle) at slightly higher sensitivity and specificity (80%–90%). Another study set a cut‐off value of 15% (87.5% sensitivity, 93.75% specificity, AUC 0.97) to identify elevated phasic chin EMG activity in narcolepsy patients (Dauvilliers et al., 2007).

These differences in EMG activity may be attributed to methodological variances of signal acquisition and analysis as well as small sample sizes, which needs to be explored in follow‐up investigations in larger cohorts in order to standardise threshold values for clinical practice.

By dividing polysomnographic recordings into the first and second half of the night, we could examine the temporal distribution of RWA throughout the night among the study groups. In RBD patients, EMG activity significantly increased from the first to the second half of the night, which is in line with previous studies stating that EMG activity, especially when associated with violent behaviours, is more prominent later in the night in these patients (Cipolli et al., 2011; Sasai‐Sakuma et al., 2014).

In narcolepsy patients, a moderate elevation of EMG activity remained constant throughout the night, confirming results of a previous study (Cipolli et al., 2011). It should be noted, however, that an increase in RWA in narcolepsy in the progression of the night have also been described (Buskova et al., 2009; Vanková et al., 2001). Interestingly, an increase of EMG activity throughout the night was also observed in our control group. To our knowledge, there are no studies linking RWA increase throughout the night with insomnia or NREM‐parasomnias, which formed our control group. However, a moderate temporal increase of RWA has previously been demonstrated in healthy subjects without sleep complaints (Vanková et al., 2001).

Comparisons of the occurrence and distribution of RWA, along with validated cut‐off values, allow a better understanding of this important aspect, which manifests in different ways in RBD and narcolepsy patients. Quantification through polysomnography is necessary to better understand why RWA usually occurs in a less extensive form, varies strongly between narcolepsy patients, and is distributed more evenly throughout the night, compared with RBD patients. These distinguishing factors might also play an important role in further investigating pathophysiological mechanisms underlying both conditions, that remain to be elucidated (Antelmi et al., 2019; Antelmi et al., 2020; Cipolli et al., 2011; Franceschini et al., 2011).

### 4.1 Limitations

The scarcity of disorders such as narcolepsy in the general population along with the retrospective design of the study led to relatively small sample sizes of our subgroups, which are, however, comparable to previous investigations (Khalil et al., 2013). Nevertheless, due to the small size of the study, the results must be interpreted with caution. Patients in the RBD subgroup included isolated and symptomatic RBD cases, a stratification, however, was not possible due to the small sample size. Age and gender distribution might therefore not be representative, however, we found that, aligning with previous studies, the RBD group had a higher prevalence of male patients, while the gender distribution was more balanced or showed a higher prevalence of female patients in the narcolepsy group (Scheer et al., 2019; Zhang et al., 2008). Regarding age, RBD‐patients were typically older than narcolepsy patients, as in previous studies (Feketeova et al., 2020; Nightingale et al., 2005). Three patients with narcolepsy and four patients with RBD reported the use of antidepressants, which has previously been linked to increased RWA, particularly when patients were taking SSRIs and SNRIs (Ferri et al., 2021; Lee et al., 2016).

The RBD patients of this study had a significantly higher AHI rates compared with the other groups, with three RBD patients being diagnosed with mild sleep apnea. Although the total AHI was higher in the RBD group, there was no significant difference in REM‐sleep related AHI, which suggests that sleep apnea had no influence on the elevation of EMG activity in REM sleep. AHI in NREM‐sleep was, however, significantly higher in RBD patients than in narcolepsy patients or in controls.

The small number of recorded muscles might be another limitation of this study. The recording of the mentalis and tibialis anterior muscles for RBD detection has frequently been used in past studies, however, it has previously been suggested that lower limb muscle activity may also occur due to motor events unrelated to RWA such as PLM or myoclonic jerks (Frauscher et al., 2012). The additional recording of upper limb muscles such as the flexor digitorium superficialis and extensor digitorium brevis muscles has previously been suggested to yield high discriminative power for the detection of RBD (Frauscher et al., 2012; Iranzo et al., 2011). Future studies might therefore include additional EMG channels for further examination of REM‐sleep related motor behaviour and differentiation from NREM‐related EMG activity and breathing artefacts.

### 4.2 Conclusion

Investigating REM‐associated muscle activity of patients affected by RBD or narcolepsy is of great diagnostic value and enables a better understanding for the pathophysiology of these sleep disorders. The quantification of RWA can provide highly sensitive and specific cut‐off values for diagnosis, with particularly high diagnostic accuracy for RBD. In narcolepsy patients, the recognition of RWA as a common and important feature and the analysis of REM‐sleep associated muscle activity could help to clarify the diagnosis and counteract diagnostic delay, especially in patients with narcolepsy type 2, who lack the characteristic clinical feature of cataplexy. Increasing RWA throughout the night with explicitly high levels of EMG activity in the second night half is characteristic for RBD, while in narcolepsy patients, an evenly distributed, moderately increased muscle activity during REM sleep might be of future diagnostic value. Future longitudinal studies in larger samples are needed to confirm these findings and to standardise threshold values for diagnostic criteria in these sleep disorders.

## AUTHOR CONTRIBUTIONS

**Franziska Edlinger:** Conceptualization; writing – original draft; writing – review and editing. **Marion Böck:** Resources; writing – review and editing. **Stefan Seidel:** Conceptualization; project administration; supervision; validation; writing – review and editing. **Karin Trimmel:** Conceptualization; project administration; supervision; validation; writing – review and editing.

## FUNDING INFORMATION

None declared.

## CONFLICT OF INTEREST STATEMENT

The authors declare no conflicts of interest.

## Supporting information


**FIGURE S1.** ROC‐curves and boxplot representations of muscle activity in the mentalis (a), tibialis anterior muscle (b), and combination (c), in RBD patients and controls excluding patients taking antidepressants.
**FIGURE S2.** ROC‐curves and boxplot representations of muscle activity in the mentalis (a), tibialis anterior muscle (b), and combination (c), in narcolepsy patients and controls excluding patients taking antidepressants.

**TABLE S1.** Polysomnographic characteristics in REM‐sleep behaviour disorder, narcolepsy patients, and controls.
**TABLE S2.** ROC characteristics in REM‐sleep behaviour disorder and narcolepsy patients, excluding patients taking antidepressants.
**TABLE S3.** REM‐sleep associated EMG activity in REM‐sleep behaviour disorder, narcolepsy patients, and controls, excluding patients taking antidepressants.
**TABLE S4.** Quade's covariance analysis: Differences in REM‐sleep associated EMG activity in REM‐sleep behaviour disorder, narcolepsy patients, and controls.
**TABLE S5.** Quade's covariance analysis: differences in overnight distribution of REM‐associated EMG activity in the night halves in REM‐sleep behaviour disorder, narcolepsy patients, and controls.

## Data Availability

The data that support the findings of this study are available on request from the corresponding author. The data are not publicly available due to privacy or ethical restrictions.
