# Supplementary material for: Loss of rapid eye movement atonia in rapid eye movement sleep behaviour disorder and narcolepsy
Source: J Sleep Res. 2024 Aug 21;34(1):e14322. doi: 10.1111/jsr.14322 (PMC11744237; doi:10.1111/jsr.14322)
Supplement: Supplementary file 1 — FIGURE S1. ROC‐curves and boxplot representations of muscle activity in the mentalis (a), tibialis anterior muscle (b), and combination (c), in RBD patients and controls excluding patients taking antidepressants. FIGURE S2. ROC‐curves and boxplot representations of muscle activity in the mentalis (a), tibialis anterior muscle (b), and combination (c), in narcolepsy patients and controls excluding patients taking antidepressants. [file JSR-34-e14322-s001.docx]

Figure S1: ROC-curves and boxplot representations of muscle activity in the mentalis-(A), tibialis anterior muscle (B) and combination (C) in RBD patients and controls excluding patients taking antidepressants

Figure S2: ROC-curves and boxplot representations of muscle activity in the mentalis-(A), tibialis anterior muscle (B) and combination (C) in narcolepsy patients and controls excluding patients taking antidepressants
