# Supplementary material for: Loss of rapid eye movement atonia in rapid eye movement sleep behaviour disorder and narcolepsy
Source: J Sleep Res. 2024 Aug 21;34(1):e14322. doi: 10.1111/jsr.14322 (PMC11744237; doi:10.1111/jsr.14322)
Supplement: Supplementary file 2 — TABLE S1. Polysomnographic characteristics in REM‐sleep behaviour disorder, narcolepsy patients, and controls. TABLE S2. ROC characteristics in REM‐sleep behaviour disorder and narcolepsy patients, excluding patients taking antidepressants. TABLE S3. REM‐sleep associated EMG activity in REM‐sleep behaviour disorder, narcolepsy patients, and controls, excluding patients taking antidepressants. TABLE S4. Quade's covariance analysis: Differences in REM‐sleep associated EMG activity in REM‐sleep behaviour disorder, narcolepsy patients, and controls. TABLE S5. Quade's covariance analysis: differences in overnight distribution of REM‐associated EMG activity in the night halves in REM‐sleep behaviour disorder, narcolepsy patients, and controls. [file JSR-34-e14322-s002.docx]

|  | **RBD**  **n=16** | **Narcolepsy**  **n=15** | **Controls**  **n=19** | **p** |
| --- | --- | --- | --- | --- |
| SPT^a^ | 438.2± 46.6 | 445.8 ± 51.6 | 442.7 ± 37.2 | 0.705 |
| TIB^a^ | 482.6 (80.4) | 474 (45) | 467.4 (52.7) | 0.560 |
| WASO^a^ | 104 (97.5) | 40 (61.5) | 42,3 (42.5) | 0.001 |
| Sleep efficiency^b^ | 70.8 ± 11.1 | 85.4 ± 9.4 | 85.9 ± 9.2 | <0.001 |
| Sleep latency^a^ | 15.5 (11.8) | 5.5 (19.5) | 10 (16) | 0.68 |
| REM latency^a^ | 127.1 ± 72.6 | 60.8 ± 55.2 | 107.7± 45.2 | 0.013 |
| Sleep stage 1^b^ | 21.5 (12.5) | 14.5 (13.8) | 10.9 (5.2) | <0.001 |
| Sleep stage 2^b^ | 50.4 ± 9.9 | 47.5 ± 11.3 | 52.4 ± 5.5 | 0.305 |
| Sleep stage 3^b^ | 4.3 (8.3) | 20 (12.6) | 17.2 (7.8) | <0.001 |
| REM stage^b^ | 18.1 ± 5.3 | 16.9 ± 6.4 | 20.1 ± 4.4 | 0.115 |
| TST^a^ | 326.3 ± 49.4 | 397.7 ± 50.7 | 390.7 ± 54.1 | <0.001 |
| AHI total | 3.7 (4.6) | 0.2 (1.5) | 0.1 (0.5) | 0.019 |
| AHI REM sleep | 0 (0) | 0 (1.7) | 0 (0.7) | 0.547 |
| AHI NREM sleep | 4.2 (6) | 0 (2) | 0 (0.4) | 0.017 |
| Arousal Index Total | 17.1 ± 9.1 | 15.8 ± 7.9 | 10.7 ± 5.8 | 0.042 |
| PLM-Index Total | 23.9 (27.9) | 2.3 (14.3) | 0 (1.3) | <0.001 |
| Data are represented as mean ± SD or median (IQR) according to data distribution, SPT: Sleep period time, TIB: Time in bed, WASO: Wake after sleep onset, TST: Total sleep time, AHI: Apnoea-Hypopnea Index, PLM-Index: Periodic Limb Movement-Index, a. data in minutes, b. data in percent (%) | | | | |

Table S1: Polysomnographic characteristics in RBD, narcolepsy patients and controls

Table S2: ROC characteristics in RBD and narcolepsy patients, excluding patients taking antidepressants

Table S3: REM-sleep associated EMG activity in RBD, narcolepsy patients and controls, excluding patients taking antidepressants

|  | | **RBD**  **n=11** | **Narcolepsy**  **n=13** | **Controls**  **n=18** | **p** |
| --- | --- | --- | --- | --- | --- |
| Mentalis^a^ |  | 35.7 (36.7) | 10.7 (13.4) | 4.5 (5.4) | <0.001 |
| Tibialis ant^a^ |  | 12.7 (9.1) | 5.8 (6.8) | 2.8 (2.4) | <0.001 |
| m&t^a^ |  | 47.3 (36.4) | 16.8 (16.1) | 6.6 (6.2) | <0001 |
| Data are represented as mean ±SD, median (IQR) or frequency/percentage according to data distribution, BMI: Body-Mass-Index, a. Data in percent (%), RBD = REM-sleep behaviour disorder, tibialis ant = m. tibialis anterior, m&t = m. mentalis and m. tibialis anterior | | | | | |

|  | **RBD**  **n=11** | | | **Narcolepsy**  **n=13** | | |
| --- | --- | --- | --- | --- | --- | --- |
|  | **m** | **t** | **m & t** | **m** | **t** | **m & t** |
| Sensitivity | 100% | 90.9% | 100% | 61.5% | 69.2% | 84.6% |
| Specificity | 94.4% | 88.9% | 94.4% | 72.2% | 77.8% | 66.7% |
| Cut-off | ≥ 10.57% | ≥ 6.39% | ≥ 17.07% | ≥ 5.97% | ≥ 4.03% | ≥ 8.37% |
| 95% CI | 0.964 - 1 | 0.897 - 1 | 0.979 - 1 | 0.579 – 0.934 | 0.548 – 0.922 | 0.661 – 0.972 |
| AUC | 0.990 | 0.960 | 0.995 | 0.756 | 0.735 | 0.816 |
| RBD = REM sleep behaviour disorder, CI = confidence interval, AUC = area under the curve, m = m. mentalis, t = m. tibialis anterior, m & t = m. mentalis & m. tibialis anterior | | | | | | |

Table S4: Quade’s covariance analysis: Differences in REM-sleep associated EMG activity in RBD-, narcolepsy patients and controls

|  | | **RBD (n=11) vs. Narcolepsy (n=13)** | **Narcolepsy (n=13) vs. Controls (n=18)** | **RBD (n=11) vs. Controls (n=18)** |  |
| --- | --- | --- | --- | --- | --- |
| Mentalis^a^ |  | 0.235 | 0.002 | <0.001 |  |
| Tibialis ant^a^ |  | 0.966 | 0.002 | 0.003 |  |
| m&t^a^ |  | 0.641 | <0.001 | <0.001 |  |
| a. Data are represented as p-values, RBD = REM-sleep behaviour disorder, tibialis ant = m. tibialis anterior, m&t = m. mentalis and m. tibialis anterior | | | | | |

Table S5: Quade’s covariance analysis: differences in overnight distribution of REM-associated EMG activity in the night halves in RBD-, narcolepsy patients and controls

|  | **RBD**  **n=11** | | **Narcolepsy**  **n=13** | **Controls**  **n=18** | |
| --- | --- | --- | --- | --- | --- |
| % EMG activity first vs. second night half^a^ | 0.036 | 0.001 | | | 0.013 |
| a. Data are represented as p-values | | | | | |
